# Opposite Effect of Mast Cell Stabilizers Ketotifen and Tranilast on the Vasoconstrictor Response to Electrical Field Stimulation in Rat Mesenteric Artery

**DOI:** 10.1371/journal.pone.0073232

**Published:** 2013-08-20

**Authors:** Esther Sastre, Laura Caracuel, Fabiano E. Xavier, Gloria Balfagón, Javier Blanco-Rivero

**Affiliations:** 1 Departamento de Fisiología, Facultad de Medicina, Universidad Autónoma de Madrid, Madrid, Spain; 2 Instituto de Investigación la Paz (IdIPaz), Madrid, Spain; 3 Departamento de Fisiologia and Farmacologia, Centro de Ciências Biológicas, Universidade Federal de Pernambuco, Recife, Brazil; Center for Cancer Research, National Cancer Institute, United States of America

## Abstract

**Objectives:**

We analyzed whether mast cell stabilization by either ketotifen or tranilast could alter either sympathetic or nitrergic innervation function in rat mesenteric arteries.

**Methods:**

Electrical field stimulation (EFS)-induced contraction was analyzed in mesenteric segments from 6-month-old Wistar rats in three experimental groups: control, 3-hour ketotifen incubated (0.1 αmol/L), and 3-hour tranilast incubated (0.1 mmol/L). To assess the possible participation of nitrergic or sympathetic innervation, EFS contraction was analyzed in the presence of non-selective nitric oxide synthase (NOS) inhibitor L-NAME (0.1 mmol/L), α-adrenergic receptor antagonist phentolamine (0.1 µmol/L), or the neurotoxin 6-hydroxydopamine (6-OHDA, 1.46 mmol/L). Nitric oxide (NO) and superoxide anion (O_2_^.-^) levels were measured, as were vasomotor responses to noradrenaline (NA) and to NO donor DEA-NO, in the presence and absence of 0.1 mmol/L tempol. Phosphorylated neuronal NOS (P-nNOS) expression was also analyzed.

**Results:**

EFS-induced contraction was increased by ketotifen and decreased by tranilast. L-NAME increased the vasoconstrictor response to EFS only in control segments. The vasodilator response to DEA-NO was higher in ketotifen- and tranilast-incubated segments, while tempol increased vasodilator response to DEA-NO only in control segments. Both NO and O_2_^-^ release, and P-nNOS expression were diminished by ketotifen and by tranilast treatment. The decrease in EFS-induced contraction produced by phentolamine was lower in tranilast-incubated segments. NA vasomotor response was decreased only by tranilast. The remnant vasoconstriction observed in control and ketotifen-incubated segments was abolished by 6-OHDA.

**Conclusion:**

While both ketotifen and tranilast diminish nitrergic innervation function, only tranilast diminishes sympathetic innnervation function, thus they alter the vasoconstrictor response to EFS in opposing manners.

## Introduction

Arterial tone is regulated by the central nervous system, perivascular innervation and myogenic mechanisms, as well as by endothelial and humoral factors. Perivascular innervation plays a principal role in the regulation of vascular tone, specifically in certain vessels such as the mesenteric vascular bed [1,2], where blood flow is approximately 20-30% of the total cardiac output [3]. Inadequate mesenteric blood flow and tissue perfusion can produce relevant haemodynamic changes [3–5]. This regulation involves sympathetic and nitrergic innervations (1,2), which mainly release mainly noradrenaline (NA) or ATP from sympathetic nerve terminals [6], and nitric oxide (NO) from nitrergic innervation [7,8].

An interaction between mast cells and nerve endings has been observed in the gastrointestinal tract [9] and cardiac tissue [10]. When stimulated, these cells degranulate, subsequently releasing a variety of mediators, such as neutral proteases, growth factors, cytokines and chemokines, as well as vasoactive substances like serotonin, histamine, leukotrienes and prostaglandins [11,12], which can affect perivascular mesenteric innervation function.

Ketotifen and tranilast are effective mast cell stabilizer agents widely used in the management of allergic and inflammatory disorders. Both drugs block a calcium channel essential for mast cell degranulation, thereby stabilizing the cell membrane[12–15]. . Additionally, ketotifen and tranilast can also induce different additional effects apart from mast cell stabilization, effects that could induce different alterations in perivascular nerve function [16–18].

Based on these considerations, the aim of this study was to analyze the possible different effects of ketotifen and tranilast on sympathetic and nitrergic function in rat mesenteric arteries, as well as the mechanism/s implicated.

## Methods

### Ethics Statement

All animals were housed in the Animal Facility of the Universidad Autónoma de Madrid (Registration number EX-021U) in accordance with directives 609/86 of the E.E.C., R.D. 233/88 of the Ministerio de Agricultura, Pesca y Alimentación of Spain, and Guide for the Care and Use of Laboratory Animals published by the USA National Institutes of Health (NIH publication No. 85.23, revised 1985). The experimental protocol was approved by the Ethics Committee of the Universidad Autónoma de Madrid.

### Animals

We used 6 month-old male Wistar rats. Rats were sacrificed by CO_2_ inhalation followed by decapitation; the first branch of the mesenteric artery was carefully dissected out, cleaned of connective tissue and placed in Krebs–Henseleit solution (KHS, in mmol/L : NaCl 115, CaCl_2_ 2.5, KCl 4.6, KH_2_PO_4_ 1.2, MgSO_4_. 7H_2_O 1.2, NaHCO_3_ 25, glucose 11.1, Na _2_EDTA 0.03) at 4°C. Some samples were immediately frozen in liquid nitrogen and stored at -70 ^°^C.

### Perivascular mast cell detection

Mesenteric arteries were fixed in 4% formaldehyde in phosphate buffered saline solution (PBS, pH=7.4) for 1 hour, cryoprotected with 30% w/v sucrose in PBS (overnight), transferred to a cryomold containing Tissue-Tek OCT embedding medium (20 min) and then immediately frozen in liquid nitrogen. All samples were kept at -70 ^°^C until the day of the experiments. Frozen tissue segments were cut into 10 µm thick sections, placed on glass slides and stained with 0.1% Toluidine Blue (3 min) for perivascular mast cell detection. Sections were coverslipped and light microscopy images were taken (Nikon Eclipse TE2000-S (inverted microscope), Nikon DXM1200F (digital camera)).

### Vascular Reactivity

The method used for isometric tension recording has been described in full elsewhere [8,19]. Briefly, two parallel stainless steel pins were introduced through the lumen of the vascular segment: one was fixed to the bath wall, and the other connected to a force transducer (Grass FTO3C; Quincy, Mass., USA); in turn, this was connected to a model 7D Grass polygraph. For EFS experiments, segments were mounted between two platinum electrodes 0.5 cm apart and connected to a stimulator (Grass, model S44) modified to supply the appropriate current strength. Segments were suspended in an organ bath containing 5 mL of KHS at 37°C continuously bubbled with a 95% O_2_ -5% CO_2_ mixture (pH 7.4). Some experiments were performed in endothelium-denuded segments to eliminate the main source of vasoactive substances, including endothelial NO. This avoided possible actions by different drugs on endothelial cells that could lead to misinterpretation of results. Endothelium was removed by gently rubbing the luminal surface of the segments with a thin wooden stick. The segments were subjected to a tension of 0.5 g, which was readjusted every 15 min during a 90-min equilibration period before drug administration. After this, the vessels were exposed to 75 mmol/L KCl to check their functional integrity. Endothelium removal did not alter the contractions elicited by KCl. After a washout period, the presence/absence of vascular endothelium was tested by the ability of 10 µmol/L acetylcholine (ACh) to relax segments precontracted with 1 µmol/L noradrenaline (NA).

Frequency-response curves to EFS (1, 2, 4, 8 and 16 Hz) were performed. The parameters used for EFS were 200 mA, 0.3 ms, 1–16 Hz, for 30 s with an interval of 1 min between each stimulus, the time required to recover basal tone. A washout period of at least 1 h was necessary to avoid desensitization between consecutive curves. Three successive frequency-response curves separated by 1-hour intervals were performed in every segment. EFS responses in the presence of mast cell stabilizers ketotifen (1 µmol/L, 0.1 µmol/L or 10 nmol/L), or tranilast (1 mmol/L, 0.1 mmol/L or 10 µmol/L) were performed to evaluate the possible effect of these drugs on the neural control of vasomotor tone. To analyze a possible time-dependent effect, either ketotifen or tranilast were added to the bath for different incubation periods: 1, 2, and 3 h, before the corresponding frequency-response curves. To evaluate whether the EFS-induced contractile response had a neural origin, the blocker for nerve impulse propagation tetrodotoxin (TTX, 0.1 µmol/L) was added to the bath 30 min in advance.

Vasodilator response to ACh (0.1 nmol/L-10 µmol/L) was tested in endothelium-intact arteries from all experimental groups.

To determine the participation of NO in the EFS-induced response in all experimental groups, 0.1 mmol/L N ^G^-nitro-L-arginine methyl ester (L-NAME), the unspecific nitric oxide synthase (NOS) inhibitor, or 1 µmol/L 1400W, the specific inducible NOS inhibitor, were added to the bath 30 min before performing the frequency–response curve.

To determine the participation of adrenergic component of sympathetic innervation on the EFS-induced response in control and ketotifen- or tranilast-incubated segments, 1 µmol/L phentolamine, an α-adrenoceptor antagonist, was added to the bath 30 min before performing the frequency-response curve.

The method to deplete sympathetic innervation has been used previously by our group in this artery [20]. Briefly, control, and ketotifen-incubated endothelium-denuded mesenteric segments were incubated at room temperature for 10 minutes in KHS (NaHCO_3_ and NaH_2_PO_4_ were omitted, unbuffered solution) containing 0.02 mmol/L glutathione and 1.46 mmol/L of the neurotoxin 6-hydroxydopamine (6-OHDA). The pH of this solution was adjusted to 4.9 with 0.05 mmol/L NaOH and then the solution was covered with paraffin oil. Subsequently, the arteries were immersed in normal KHS and EFS-induced contraction experiments were performed.

The vasoconstrictor response of exogenous NA (1 nmol/L-10 µmol/L), and the vasodilator response to the NO donor diethylamine NONOate (DEA-NO, 0.1 nmol/L–0.1 mmol/L) were tested in mesenteric segments from all experimental groups. The possible participation of superoxide anions (O_2_^.-^) in the vasodilator response to DEA-NO was tested by incubation with 0.1 mmol/L of the O_2_^. -^ scavenger tempol.

### Histamine and noradrenaline release

To measure histamine and NA release, we used a Histamine Enzyme Immunoassay kit (Spibio, Berlin) and a Noradrenaline Research EIA (Labor Diagnostica Nord, Gmbh & Co., KG), respectively. Endothelium-denuded segments from control, ketotifen-incubated (0.1 µmo/L, 3 hours) or tranilast-incubated (0.1 mmo/L, 3 hours) mesenteric arteries were preincubated in 5 mL of KHS at 37°C and continuously gassed with a 95% O_2_-5% CO_2_ mixture (stabilization period). This was followed by two washout periods of 10 min in a bath of 0.4 mL of KHS. Then the medium was collected to measure basal histamine or NA release. Next, the organ bath was refilled, and cumulative EFS periods of 30 s at 1, 2, 4, 8 and 16 Hz at 1 min intervals were applied. Afterwards, the medium was collected to measure EFS-induced histamine or NA release. The assay was performed following the manufacturer’s instructions. Results were expressed as nmol Histamine /mL mg tissue or ng NA/mL mg tissue.

### Nitric Oxide Release

Nitric oxide release was determined using the fluorescent probe 4,5-diaminofluorescein (DAF-2), as previously described [21]. Briefly, endothelium-denuded arteries were divided into several experimental groups: control, and segments incubated with ketotifen (0.1 µmol/L, 3 hours), tranilast (0.1 mmol/L, 3 hours), loratadine (1 µmol/L, 30 min) or famotidine (1 µmol/L, 30 min). After an equilibration period of 60 min in HEPES buffer (in mmol/L: NaCl 119; HEPES 20; CaCl_2_ 1.2; KCl 4.6; MgSO_4_ 1; KH_2_PO_4_ 0.4; NaHCO_3_ 5; glucose 5.5; Na_2_HPO_4_ 0.15; pH 7.4) at 37^°^C, arteries were incubated with 2 µmol/L DAF-2 for 45 min. Then the medium was collected to measure basal NO release. Once the organ bath was refilled, cumulative EFS periods of 30 s at 1, 2, 4, 8 and 16 Hz at 1 min intervals were applied. The fluorescence of the medium was measured at room temperature using a spectrofluorimeter (LS50 PerkinElmer Instruments, FL WINLAB Software) with excitation wavelength set at 492 nm and emission wavelength at 515 nm.

The EFS-induced NO release was calculated by subtracting basal NO release from that evoked by EFS. Also, blank samples were collected in the same way from segment-free medium in order to subtract background emission. Some assays were performed in the presence of 0.1 µmol//L TTX, 0.1 mmol/L L-NAME or 0.1 mmol/L 7-NI, a specific nNOS inhibitor, to ensure the specificity of the method. The amount of NO released was expressed as arbitrary units/mg tissue.

### nNOs and P-nNOS expression

Western blot analysis of nNOS and phosphorylated nNOS (P–NOS) expression was performed as previously described [22,23]. Rabbit polyclonal antibody against nNOS (1:1000 dilution, Abcam), rabbit polyclonal antibody against P-nNOS (1:1000 dilution, Abcam), and monoclonal anti-β-actin-peroxidase antibody (1:50000, Sigma-Aldrich, Spain) were used. Rat brain homogenates were used as a positive control.

### Detection of O_2_^-^

O_2_^-^ levels were measured using lucigenin chemiluminescence, as previously described [22,24]. Briefly, endothelium-denuded segments of control, ketotifen-incubated (0.1 µmo/L, 3 hours) or tranilast-incubated (0.1 mmo/L, 3 hours) mesenteric arteries were equilibrated for 30 min in HEPES buffer at 37^°^C, transferred to test tubes that contained 1 mL HEPES buffer (pH 7.4) containing lucigenin (5 µmol/L) and then kept at 37 ^°^C. The luminometer was set to report arbitrary units of emitted light; repeated measurements were collected during 5 min at 10 s intervals and averaged. 4,5-Dihydroxy-1,3-benzene-disulphonic acid ‘‘Tiron’’ (10 mmol/L), a cell permeant, non-enzymatic O_2_^-^ scavenger^-^, was added to quench the O_2_^-^-dependent chemiluminescence. Some segments were preincubated with 0.1 mmol/L tempol before the experimental protocol was performed, in order to ensure the specificity of the method. Also, blank measures were collected in the same way without mesenteric segments to subtract background emission.

### 3-nitrotyrosine (3-NT) detection

3-NT levels were determined using the Nitrotyrosine ELISA kit from Abcam (Cambridge, UK). For this assay, frozen endothelium-denuded segments of control, ketotifen-incubated (0.1 µmo/L, 3 hours) or tranilast-incubated (0.1 mmo/L, 3 hours) mesenteric arteries were homogenized in PBS and centrifuged at 600g for 10 min at 4^°^C. The supernatant was then collected and used for the assay. 3-NT was measured following the manufacturer’s protocol. Results were normalized with protein content, using a DC protein assay kit (Bio-Rad Laboratories, Hercules, CA, USA). Results are expressed as ng 3-NT/mg protein.

### Drugs used

L-NA hydrochloride, 6-hydroxydopamine (6-OHDA), ACh chloride, diethylamine NONOate diethylammonium salt, TTX, 1400W, L -NAME hydrochloride, 7- nitroindazole, tempol, phentolamine, DAF-2, lucigenin and tiron were purchased from Sigma-Aldrich (Spain). Stock solutions (10 mmol/L) of drugs were made in distilled water, except for NA, which was dissolved in NaCl (0.9%)-ascorbic acid (0.01% w/v), and 7NI and tempol, which were dissolved in DMSO. These solutions were kept at -20°C and appropriate dilutions were made in KHS on the day of the experiment.

### Data Analysis

The responses elicited by EFS or NA were expressed as a percentage of the initial contraction elicited by 75 mmol/L KCl for comparison between control, ketotifen-incubated and tranilast-incubated segments. The relaxation induced by DEA-NO was expressed as a percentage of the initial contraction elicited by NA (Control: 995.5 + 14.6 mg; ketotifen: 1002.9 + 29.41 mg; tranilast: 946.7 + 28.47 mg, P> 0.05). Results are given as mean + S.E.M. Statistical analysis was done by comparing the curve obtained in the presence of the different substances with the previous or control curve by means of repeated measure analysis of variance (ANOVA) followed by the Bonferroni post-hoc test. Some results were expressed as differences of area under the curve (dAUC). AUC were calculated from the individual frequency-response plots. For dAUC, histamine, NO and NA release experiments, the statistical analysis was done using one-way ANOVA followed by Newman-Keuls post-hoc test. *P*< 0.05 was considered significant.

## Results

### Perivascular mast cell detection

**Figure 1:**
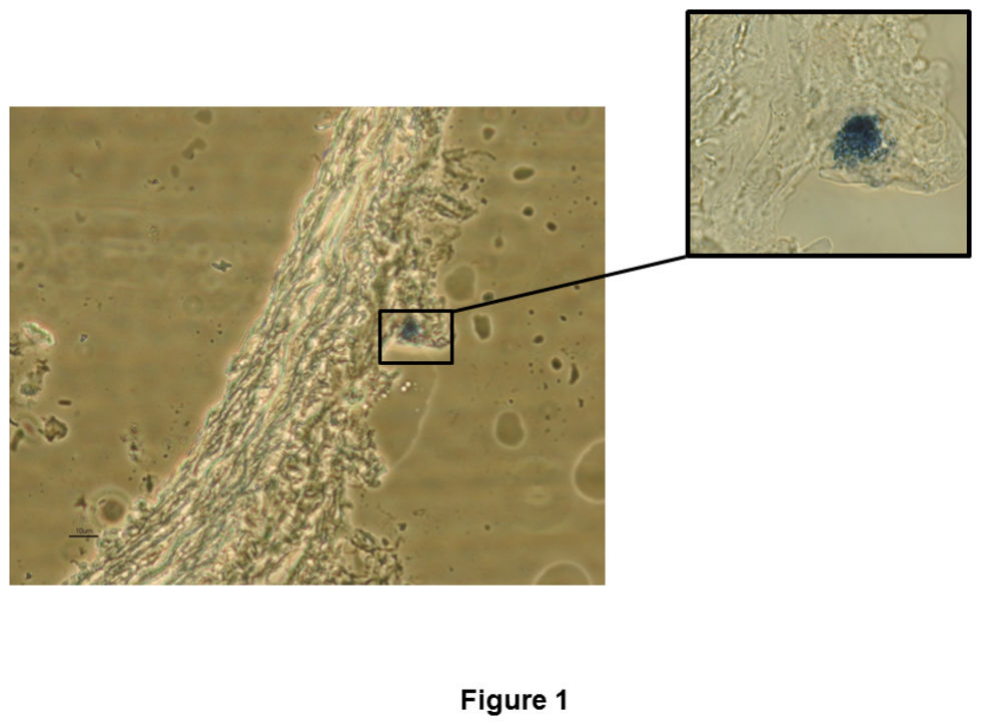
Mast cell localization by toluidine blue staining. Figure is representative of preparations from four rats. Magnification: 400·X and 600 X.

Mast cells were detected in the adventitial layer of mesenteric arteries using toluidine blue staining (Figure 1).

### Histamine release

Basal histamine release was significantly lower in ketotifen-incubated and tranilast-incubated segments than in control arteries. EFS increased histamine release in all experimental groups, but the increase was greater in control segments. Preincubation with TTX abolished EFS-induced histamine release in all experimental conditions (Table 1).

**Table 1:** Effect of 0.1 µmol/L ketotifen and 0.1 mmol/L tranilast on basal and EFS-induced histamine and NA release.

	**Histamine release**		**NA release**	
	**Basal**	**EFS**	**Basal**	**EFS**
**Control**	**0.51 + 0.15**	1.19 + 0.27*	**6.65 + 0.99**	9.25 + 1.13*
***Ketotifen***	**0.15 + 0.03^#^**	0.38 + 0.16*^#^	**6.67 + 0.60**	9.33 + 0.88*
***Tranilast***	**0.19 + 0.02^#^**	0.52 + 0.11*^#^	**6.55 + 0.65**	8.89 + 1.05*
***TTX***	**0.58 + 0.09**	**0.66 + 0.17^#^**	**6.37 + 0.74**	**6.45 + 0.87^#^**

This table presents histamine and NA levels released in basal and EFS conditions. Results (means + SEM) are expressed as nmol histamine /mL mg tissue, or ng NA/mL mg tissue. *P< 0.05 vs basal conditions. #P< 0.05 vs control. n= 6 animals each group.

### Vasomotor Response to KCl

In endothelium-intact mesenteric segments, the vasoconstrictor response to 75 mmol/l KCl was similar in all experimental groups (Control, 964.3 ± 59.9 mg, 0.1 µmol/L ketotifen, 913.6 ± 74.5 mg, 0.1 mmol/L tranilast ; 917.8 ± 65.2 mg; P > 0.05; n= 10 each group). Endothelium removal did not alter KCl-induced vasoconstriction (Control, 976.3 ± 67.6 mg, 0.1 µmol/L ketotifen, 912.8 ± 62.7 mg, 0.1 mmol/L tranilast ; 915.5 ± 54.4 mg; P > 0.05; n= 10 each group).

### Vascular responses to EFS

**Figure 2:**
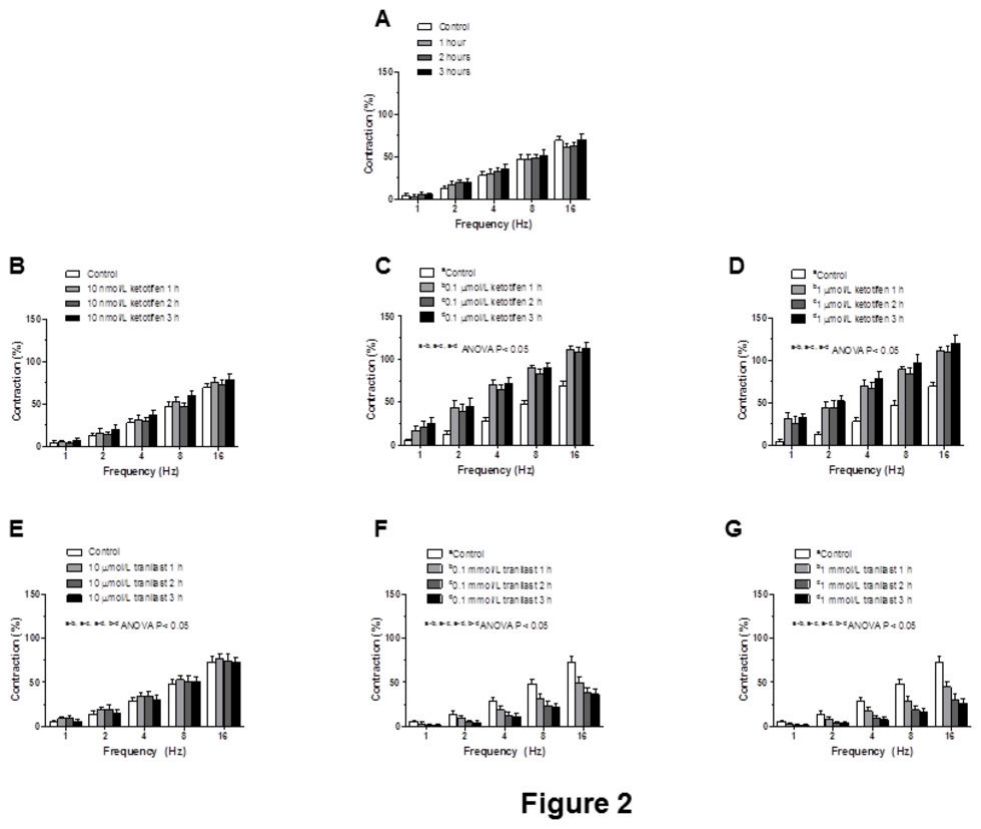
Vasoconstrictor response to EFS (A) Isometric tension recording of the frequency-dependent contractions in intact mesenteric artery segments from Wistar rats. Effect of preincubation with 10 nmol ketotifen (B), 1 µmol/L ketotifen (C), 0.1 µmol/L ketotifen (D), 10 µmol/L tranilast (E), 0.1 mmol/L tranilast (F) or 1 mmol/L tranilast (G) for 1, 2 and 3 hours on frequency dependent contraction in mesenteric segments from Wistar rats. Results (means ± S.E.M.) are expressed as a percentage of tone induced by 75 mmol/L KCl. n= 10 animals each group.

The application of EFS induced a frequency-dependent contractile response in endothelium-intact mesenteric segments. Four consecutive EFS curves were performed at 1-h intervals in control mesenteric segments, and induced similar contractions (Figure 2A). Preincubation with 10 nmol/L ketotifen did not modify EFS-induced contractions, while preincubation with 0.1 µmol/L or 1 µmol/L ketotifen for 1, 2 and 3 hours increased the contraction induced by EFS to a similar extent (Figure 2B–2D). Preincubation with 0.1 mmol/L or 1 mmol/L tranilast for 1 or 2 hours induced a progressive decrease in EFS-induced contractions that was stabilised after 3 hours. Preincubation with 10 µmol/L tranilast did not modify EFS-induced contractions (Figure 2E–2G). For this reason, we performed the following experiments preincubating with either 0.1 µmol/L ketotifen or 0.1 mmol/L tranilast during 3 hours.

**Figure 3:**
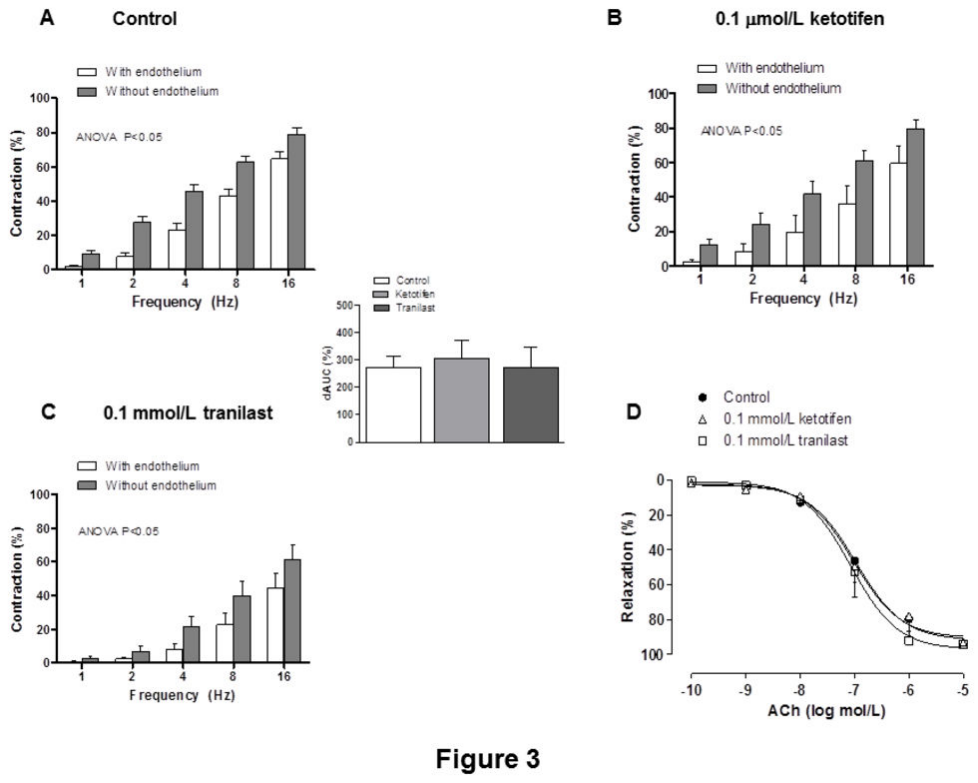
Endothelium influence on vasoconstrictor response to EFS. Effect of endothelium removal on the vasoconstrictor response to electrical field stimulation in control (A), ketotifen-incubated (B) or tranilast-incubated (C) mesenteric segments from Wistar rats. Results (means ± S.E.M.) are expressed as a percentage of tone induced by 75 mmol/L KCl. n= 10 animals each group. Insert graph shows differences of area under the curve (dAUC) in the absence or presence of 01 μmol/L phentolamine, expressed as arbitrary units. * P< 0.05 control vs. tranilast. (D) ACh-induced vasodilation in endothelium-intact control, ketotifen-incubated or tranilast-incubated mesenteric segments. Results (mean + S.E.M.) were expressed as a percentage of the previous tone elicited by exogenous NA. n = 6 animals each group.

Endothelium removal increased EFS-induced contractile response similarly in segments from all experimental groups (Figure 3A–3C). EFS-induced contractions were practically abolished in segments from all experimental groups by the blocker for nerve impulse propagation, TTX (0.1 µmol/L; table 2).

**Table 2:** Effect of preincubation with tetrodotoxin (TTX, 0.1 µmol/L) or 6-hydroxydopamine (6-OHDA, 1.46 mmol/L) on the frequency–contraction curves performed in control, ketotifen-incubated (0.1 µmol/L) and tranilast-incubated (0.1 mmol/L) mesenteric segments.

	**1 Hz**	**2 Hz**	**4 Hz**	**8 Hz**	**16 Hz**
**Control**	9.2 + 1.7	27.6 + 3.5	45.9 + 3.4	62.6 + 3.3	79.1 + 3.7
*TTX*	0	0	0	0.5 + 0.04	0.9 + 0.2
*6-OHDA*	0	0	0.1 + 0.03	0.2 + 0.05	0.4 + 0.1
**Ketotifen**	17.8 + 1.7	34.9 + 4.1	53.5 + 4.8	69.6 + 3.8	90.1 + 2.6
*TTX*	0	0	0	0.2 + 0.01	0.6 + 0.1
*6-OHDA*	0	0	0.2 + 0.06	0.5 + 0.1	0.9 + 0.2
**Tranilast**	2.5 + 1.1	7.0 + 3.1	21.3 + 6.1	39.9 + 8.2	61.5 + 8.7
*TTX*	0	0	0	0	0.1 + 0.01

Results (means ± S.E.M.) are expressed as percentages of the response elicited by 75 mM KCl; zeros are used when contraction was not detected. n = 5–7 animals.

### Vasodilator response to ACh

The vasodilator response to ACh was similar in all experimental groups (Figure 3D, table 3).

**Table 3:** E_max_ and log EC_50_ values of vasodilator responses to ACh in control, ketotifen-incubated (0.1 μmo/L) or tranilast-incubated (0.1 mmol/L) mesenteric arteries from Wistar rats.

		**E_max_**		**log EC_50_**	
**Control**		91.88 + 2.62		-6.98 + 0.09	
*Ketotifen*		97.22 + 3.83		-7.07 + 0.13	
*Tranilast*		90.64 + 3.68		-7.02 + 0.15	

Results are expressed as means + S.E.M. n=6 animals each group.

### Effect of ketotifen or tranilast on the nitrergic component of vascular responses to EFS

**Figure 4:**
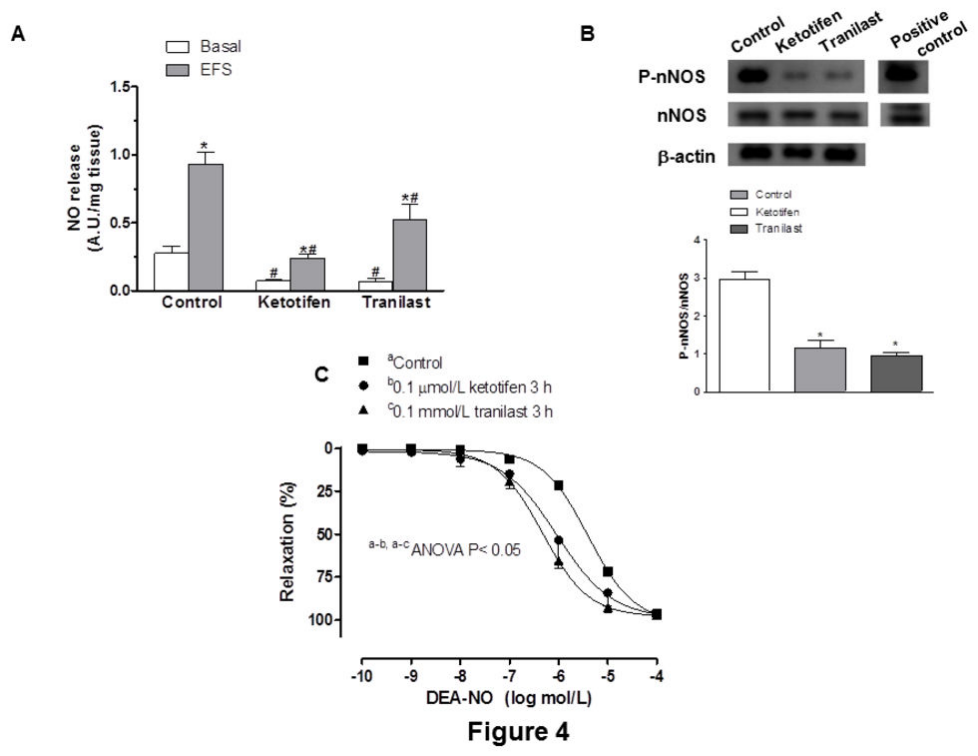
Effect of ketotifen and tranilast on neuronal NO synthesis and vasodilation (A) Effect of preincubation with ketotifen or tranilast on basal and EFS-induced NO release in mesenteric segments. n = 5 animals each group. Results (means + S.E.M.) are expressed as arbitrary units (A.U.)/mg tissue. *P< 0.05 vs basal; # P< 0.05 vs control. (B) Western blot for nNOS and P-nNOS expression in control, ketotifen- and tranilast-incubated mesenteric segments. Figure is representative of preparations from six samples in each group. Lower panel shows relation between densitometric analyses for P-nNOS vs. nNOS expression. * P< 0.05. (C) Vasodilator response to DEA-NO in control, ketotifen- incubated and tranilast-incubated mesenteric segments. Results (means + S.E.M.) are expressed as a percentage of the inhibition of contraction induced by NA. n = 6 animals each group.

Basal NO release was higher in control than in ketotifen- or tranilast-incubated mesenteric segments (Figure 4A). EFS increased NO release in all experimental groups, but the increase was greater in control arteries (Figure 4A). Preincubation with L-NAME (0.1 mmol/L), 7NI (0.1 mmol/L) or TTX (0.1 µmol/L) practically abolished EFS-induced NO release in arteries from either treatment group (table 4). Additionally, preincubation with either the H1 receptor antagonist loratadine (1 µmol/L) or the H2 receptor antagonist famotidine (1 µmol/L) for 30 minutes did not modify basal or EFS-induced NO release (table 4).

**Table 4:** Effect of preincubation with L-NAME (0.1 mmol/L), 7-nitroindazol (7-NI, 0.1 mmol/L), loratadine 7-NI, 1 mmol/L loratadine, 1 mmol/L famotidine or 0.1 mmol/L TTX on basal and EFS-induced NO release in control, ketotifen-incubated (0.1 mmol/L) and tranilast-incubated (0.1 mmol/L) mesenteric segments.

	**Basal**	**EFS**
**Control**	0.28 + 0.05	0.95 + 0.09*
*L-NAME*	0.26 + 0.04	0.29 + 0.07^#^
*7-NI*	0.27 + 0.09	0.31 + 0.06^#^
*Loratadine*	0.32 + 0.07	0.92 + 0.13*
*Famotidine*	0.27 + 0.12	0.94 + 0.17*
*TTX*	0.25 + 0.03	0.28 + 0.11^#^
**Ketotifen**	0.07 + 0.01	0.23 + 0.11*
*L-NAME*	0.05 + 0.03	0.07 + 0.04^#^
*7-NI*	0.09 + 0.04	0.11 + 0.04^#^
*TTX*	0.06 + 0.03	0.07 + 0.03^#^
**Tranilast**	0.07 + 0.02	0.38 + 0.16*
*L-NAME*	0.06 + 0.01	0.09 + 0.04^#^
*7-NI*	0.06 + 0.02	0.08 + 0.03^#^
*TTX*	0.04 + 0.01	0.04 + 0.01^#^

Results (means ± S.E.M.) are expressed in arbitrary units (A.U.)/mg tissue. n = 6-10 animals each group. *P < 0.05 compared with the respective basal NO release. #P < 0.05 compared with conditions without specific inhibitor.

The expression of nNOS was not modified by incubation with either ketotifen or tranilast (Figure 4B). P-nNOS expression was decreased in homogenates from ketotifen- or tranilast-incubated arteries compared to expression in control segment homogenates (Figure 4B).

**Figure 5:**
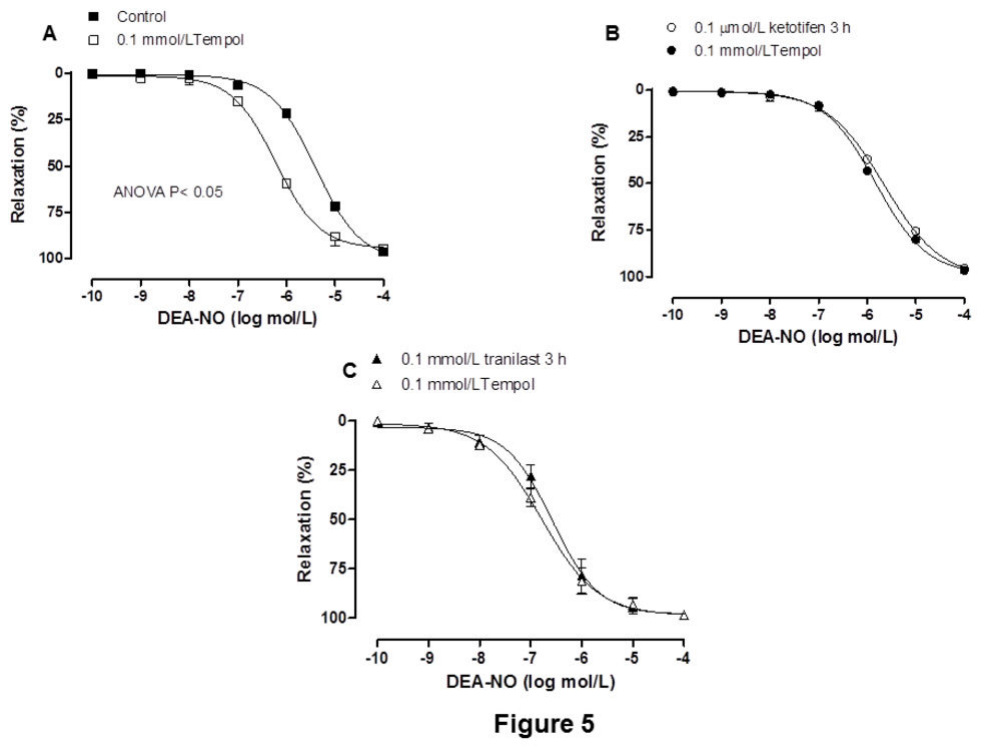
Effect of superoxide anions on NO-dependent vasodilation. **Effect of 0.1 mmol/L tempol on the concentration–response curves to DEA-NO in control (A) ketotifen-incubated (B) and tranilast-incubated (C) mesenteric segments**. Results (means + S.E.M.) are expressed as a percentage of the inhibition of the contraction induced by NA. n=6 animals each group.

In NA-precontracted mesenteric segments (Control: 995.5 + 14.6 mg; ketotifen: 1002.9 + 29.41 mg; tranilast: 946.7 + 28.47 mg), DEA-NO (0.1 nmol/L–0.1 mmol/L) induced a concentration-dependent relaxation that was greater in segments preincubated with either mast cell stabilizer than in control segments (Figure 4C, table 5). The O_2_^-^ scavenger tempol increased vasodilator response to DEA-NO in control segments, but not in ketotifen- or tranilast- incubated segments (Figure 5, table 5). Additionally, after subtracting the lucigenin chemiluminescence obtained in the presence of tiron from that obtained in its absence, the calculated tiron-quenchable chemoluminescence was significantly lower in ketotifen-incubated and tranilast-incubated segments than in control mesenteric segments. Preincubation of segments with tempol strongly decreased the tiron-quenchable chemiluminescence (Table 6). Additionally, 3-NT levels were decreased in ketotifen-incubated and tranilast-incubated segments (In ng 3-NT/mg protein: Control^a^: 1.71 + 0.31; 0.1 µmol/L ketotifen^b^: 0.92 + 0.06; 0.1 mmol/L tranilast^c^: 0.98 + 0.11; ^a-b, a-c^ P< 0.05; n = 6 animals each group).

**Table 5:** E_max_ and log EC_50_ values of vasodilator responses to DEA-NO in control, ketotifen-incubated (0.1 µmol/L) or tranilast-incubated (0.1 mmol/L mesenteric arteries from Wistar rats before and after preincubation with tempol (0.1 mmol/L).

	**Untreated**		**Tempol-treated**	
	**E_max_**	**log EC_50_**	**E_max_**	**log EC_50_**
**Control**	101.5 + 2.16	-5.39 + 0.04	94.76 + 7.52	-6.10 + 0.18^#^
*Ketotifen*	99.11 + 8.40	-6.14 + 0.18*	101.10 + 2.38	-5.95 + 0.15
*Tranilast*	98.02 + 2.44	-6.33 + 0.07*	98.62 + 3.03	-6.38 + 0.08

Results are expressed as means + S.E.M. n= 6 animals each group. *P < 0.05 vs. control segments. #P< 0.05 tempol incubated vs. unincubated segments.

**Table 6:** Effect of 0.1 µmol/L ketotifen or 0.1 mmol/L tranilast on O_2_^. -^ release. Effect of preincubation with 0.1 mmol/L tempol on O_2_^. -^ release in control, ketotifen-incubated and tranilast-incubated mesenteric segments.

	**Untreated**	**Tempol-treated**
**Control**	103.4 + 8.2	5.4 + 1.3^#^
*Ketotifen*	54.2 + 1.6*	2.1 + 1.8^#^
*Tranilast*	39.1 + 2.8*	3.1 + 1.0^#^

Results (means + S.E.M.) are expressed in chemoluminescence units/ min mg tissue. *P < 0.05 compared with control group. #P < 0.05 compared with conditions without tempol. n= 6-10 animals each group.

**Figure 6:**
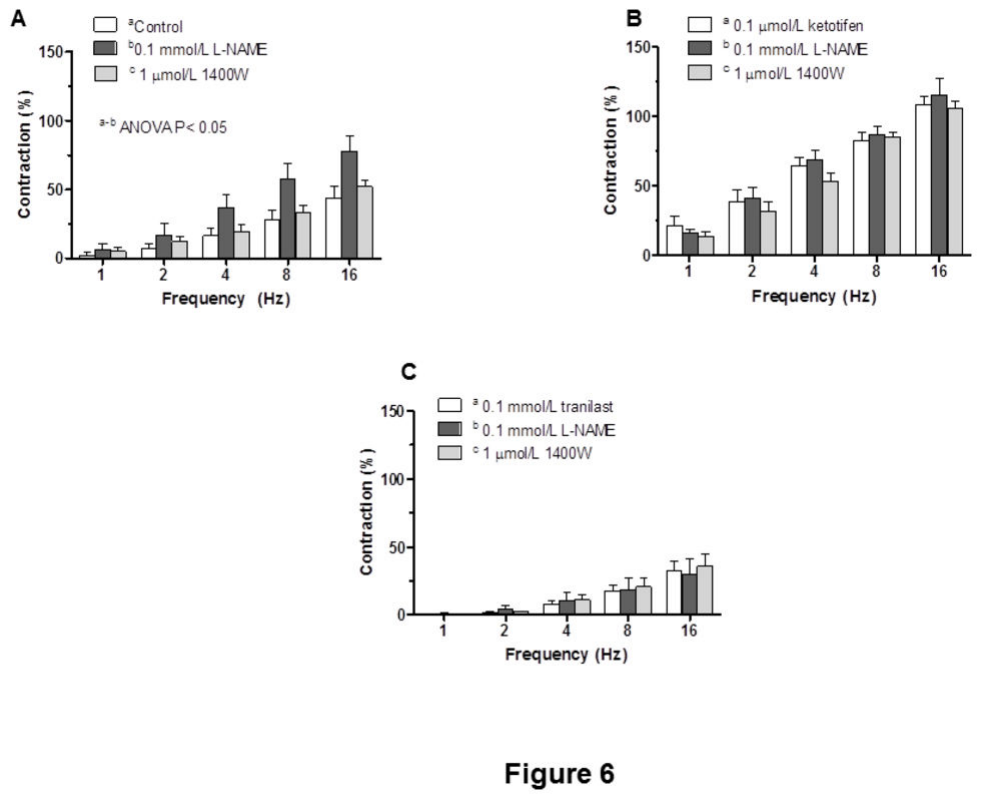
Effect of ketotifen or tranilast on nitrergic innervation function. Effect of preincubation with 0.1 mmol/L L-NAME or 1 µmol/L 1400W on the frequency-response curves in control (A) ketotifen-incubated (B) or tranilast-incubated (C) mesenteric segments. Results (means + S.E.M.) are expressed as a percentage of tone induced by 75 mmol/L KCl. n = 6 animals each group.

In line with these results, the contraction induced by EFS was significantly increased by preincubation with the unspecific NOS inhibitor L-NAME (0.1 mmol/L) in control segments, but it did not have any effect in either ketotifen- or tranilast-treated segments (Figure 6). The specific iNOS inhibitor 1400W did not modify EFS-induced contraction in any experimental group (Figure 6).

### Effect of preincubation with ketotifen or tranilast on sympathetic innervation

**Figure 7:**
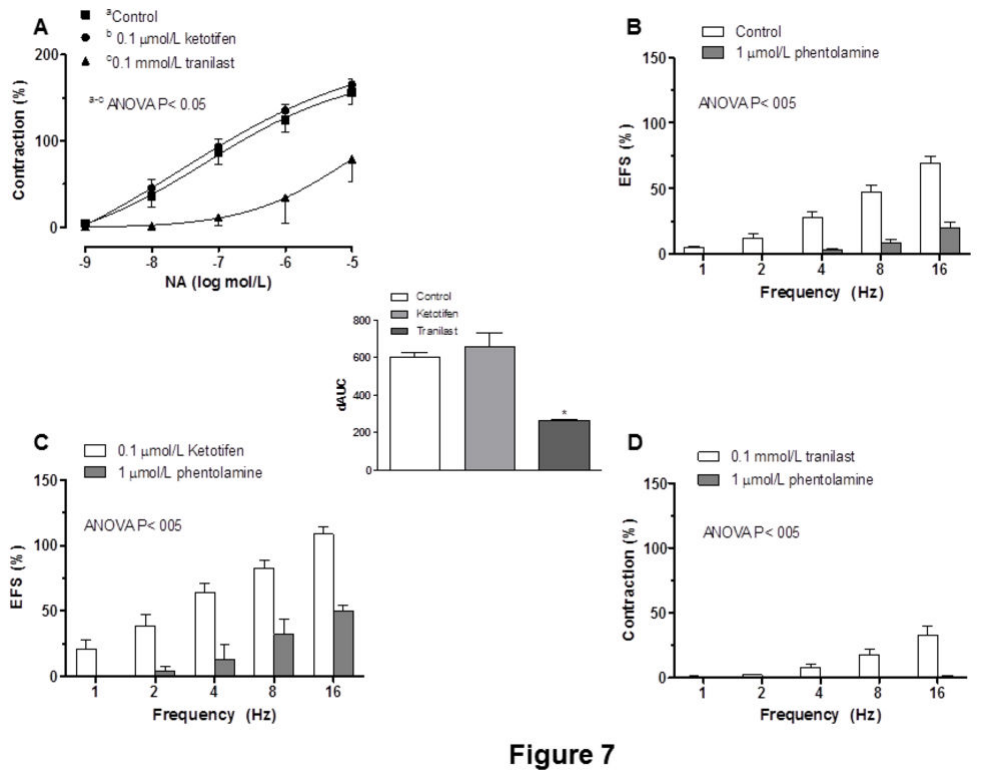
Effect of ketotifen or tranilast on sympathetic innervation function. (A) Effect of preincubation with ketotifen or tranilast on the vasocostrictor response curves to noradrenaline. Effect of 0.1 µmol/L phentolamine on the frequency-response curve in control (B), ketotifen-incubated (C) or tranilast-incubated (D) segments. Results (means + S.E.M.) are expressed as a percentage of tone induced by 75 mmol/L KCl. **n = 5-6 animals each group. Insert graph shows differences of area under the curve (dAUC) in the absence or presence of 01 μmol/L phentolamine, expressed as arbitrary units**. * **P< 0.05 control vs. tranilast**.

Preincubation with 0.1 µmol/L ketotifen did not modify the NA contractile response (0.1 nmol/L–10 µmol/L) (Figure 7A), while the response was decreased in 0.1 mmol/L tranilast-preincubated segments (Figure 7A). Both basal and EFS-induced NA releases were not modified by preincubation with either 0.1 µmol/L ketotifen or 0.1 mmol/L tranilast (Table 1). The contraction elicited by EFS was significantly reduced by the α-adrenoceptor antagonist, phentolamine (1 µmol/L), in segments from control, ketotifen-incubated and tranilast-incubated segments. The decrease was lower in tranilast-incubated than in control mesenteric segments (Figure 7B–7D). Preincubation with 6-OHDA practically abolished the EFS-induced contraction in segments from control and ketotifen-incubated mesenteric segments (Table 2).

## Discussion

The results of the present study demonstrate for the first time that the mast cell stabilizers ketotifen and tranilast modify EFS-induced vasoconstriction differently in rat mesenteric arteries. Ketotifen increased EFS-induced vasoconstriction, an effect that seems to be mediated at least by a decrease in neuronal NO release, while tranilast, although also decreasing neuronal NO release, diminished EFS-induced contraction through a decreased vasoconstrictor response to NA. The effect of these drugs on neuronal NO release is mediated by a decrease in nNOS phosphorylation.

The remarkable connection between innervation and mast cells has attracted much interest. This aspect has been widely studied in the abdominal cavity, where mast cell activation modulates various gastrointestinal functions [9,25–28]. In the current study, we have observed that mast cells are located in the adventitial layer, and that histamine is released under basal conditions indicating, that perivascular mast cells are tonically activated. Neurotransmitter release has been described to alter mast cell activation and their release of multiple mediators, including histamine [11,12]. In the current study, histamine release was enhanced by EFS. The fact that preincubation with TTX abolishes EFS-induced histamine release reveals for the first time that neurotransmitter release from mesenteric artery innervation activates mast cell degranulation. Both basal and EFS-induced histamine release were decreased by ketotifen or tranilast incubation, confirming the stabilizing role of these drugs.

The interplay of mast cells with perivascular neuronal function and the possible functional consequences are highly interesting when examining aspects of hemodynamic changes when mast cells are stabilized. Thus, the current study was designed to analyze the effect of mast cell stabilization on perivascular innervationfunction. For this purpose, we studied the effect of ketotifen and tranilast on the vasomotor response produced by EFS in rat superior mesenteric arteries.

We first analyzed whether ketotifen and tranilast modified the EFS-induced contraction observed in mesenteric segments. As we showed in the *Results* section, after performing a time-course pilot study, we considered it appropriate to perform experiments with either 0.1 µmol/L ketotifen or 0.1 mmol/L tranilast for 3 hours. EFS produced a frequency-dependent contraction in endothelium-intact mesenteric segments from all the experimental groups as reported in previous reports by our group [22,23,29,30]. We observed that ketotifen and tranilast had opposite effects: while preincubation with ketotifen increased the contraction induced by EFS, tranilast decreased that contraction. These modifications were not attributable to changes in the intrinsic contractile machinery as was demonstrated by the similar vasoconstrictor response to KCl in all experimental groups. Endothelium removal increased vasoconstrictor response to EFS to the same extent in the three experimental groups, indicating that the modulating role of endothelium is not modified by either ketotifen or tranilast. The fact that ACh-induced vasodilation was not modified in any experimental group reinforces this observation. Therefore, these results indicate that the modifications observed after preincubation with either ketotifen or tranilast are due to modifications in perivascular innervation function, as was confirmed by the abolishment of EFS-induced vasoconstriction in the presence of TTX.

Superior mesenteric artery possesses different innervations, mainly vasodilator nitrergic and vasoconstrictor sympathetic innervations, which are implicated in the control of vascular tone [6–8]. We analyzed the participation of each kind of innervation in the vasoconstriction induced by EFS, and the possible modifications in this participation that were induced by either ketotifen or tranilast.

Ketotifen and tranilast have been reported to alter eNOS and iNOS activity in several tissues [31–33]. However, to our knowledge, there are no reports about possible alterations in nNOS expression and/or activity in perivascular nerve endings. Therefore, our next objective was to analyze the possible effect of ketotifen and tranilast on neuronal NO release from nitrergic nerve endings. Preincubation with ketotifen or tranilast diminished both basal and EFS-induced NO release. The fact that, in all cases, EFS-induced NO release was abolished by preincubation with TTX, L-NAME, the non-specific NOS inhibitor, or 7-nitroindazol (7-NI), the specific nNOS inhibitor, confirms the neural origin of the NO release.

Besides its role as a mast cell membrane stabilizer, it has been clearly demonstrated that ketotifen also possesses powerful and sustained non-competitive histamine blocking properties, as it antagonizes both the H1 and H2 histamine receptors. The fact that neither the selective H1 antagonist loratadine nor the selective H2 antagonist famotidine modified either basal or EFS-induced NO release rules out the participation of histamine receptors in this response.

NO released from nerve endings is biosynthesized by nNOS [8,22,23,30]. Since preincubation with either ketotifen or tranilast decreased both basal and EFS-induced NO release, our next objective was to determine if these decreases produced by ketotifen and tranilast were due to modifications in nNOS expression and/or activity. We found that nNOS protein expression was not modified. nNOS must be phosphorylated in order to be activated. P-nNOS expression was decreased by treatment with either ketotifen or tranilast. These results indicate that the decreased NO release observed after preincubation with either ketotifen or tranilast is due to a decrease in nNOS activation. To our knowledge this is the first study to demonstrate the actions of ketotifen and tranilast on neuronal NO release from perivascular innervation in mesenteric bed.

Previously, we have demonstrated in this rat strain that both the non-selective NOS inhibitor L-NAME and the specific nNOS inhibitor 7-NI decrease EFS-induced NO release to a similar extent [30]. However, in vascular reactivity experiments, preincubation with 7-NI also decreased vasoconstrictor response to NA, making the analysis of EFS-induced contractions very complex, and leading to result misinterpretation [29,30]. For that reason, we used L-NAME in vascular reactivity experiments. L-NAME did not modify EFS-induced contractions in ketotifen or tranilast-incubated segments, in contrast to the increase observed in control segments, thus suggesting that both ketotifen and tranilast decrease nitrergic innervation function. Preincubation with the specific iNOS inhibitor 1400W did not modify vasoconstrictor response to EFS, ruling out an iNOS participation in the effects of ketotifen and tranilast.

Possible alterations in smooth muscle sensitivity to NO cannot be ruled out. A decrease in O_2_^-^ was observed in ketotifen- and tranilast-incubated mesenteric segments, in agreement with the previously described antioxidant effect of both mast cell stabilizers [34,35]. Additionally, 3-NT detection, used as a stable marker of peroxynitrite detection [36], showed a marked decrease in segments preincubated with either ketotifen or tranilast. Therefore, both ketotifen and tranilast could alter NO metabolism and bioavailability. The vasodilator response to DEA-NO was increased by preincubation with either ketotifen or tranilast. Preincubation with the O_2_^-^scavenger tempol increased vasodilator response to DEA-NO in control segments, but not in ketotifen or tranilast-incubated segments, thus confirming a decreased NO metabolism through decreased O_2_^-^release.

Therefore, ketotifen and tranilast induce two opposite effects: a decrease in neuronal NO release, and an increase in NO bioavailability due to a decrease in O_2_^-^ formation. The net effect is a decreased role for nitrergic innervation.

The fact that both ketotifen and tranilast exert the same effect on neuronal NO release does not explain why they produce opposite effects in the EFS-induced contractile response. Therefore, different influences by ketotifen and tranilast on the function of other innervations cannot be ruled out. Given the principal role played by sympathetic innervation in EFS-induced vasoconstriction [2,8], we analyzed the possible influence of ketotifen and tranilast on EFS-induced vasoconstriction. Vasoconstriction elicited by EFS was strongly reduced by phentolamine in segments from all experimental groups, indicating that this response was mediated mainly by NA release from the adrenergic component of sympathetic nerve terminals, with subsequent activation of α-adrenoceptors. This decrease was similar in control and ketotifen-incubated mesenteric segments. However, the decrease in EFS-induced contraction obtained by preincubation with phentolamine was lower in tranilast-preincubated segments, suggesting different influences by ketotifen and tranilast on the adrenergic component of sympathetic innervation. These differences could be due to modifications in either NA release or vasoconstrictor response to exogenous NA. When analysing NA release, we observed that it was not modified by mast cell stabilization with either ketotifen or tranilast. This result shows that the effect of these drugs on EFS-induced vasoconstriction is not mediated by changes in NA release. When concentration–response curves to exogenous NA were performed, we observed that ketotifen did not modify the vasoconstrictor response, while tranilast decreased it. Taken together, these results confirm that the adrenergic component of sympathetic innervation is not affected by ketotifen in the mesenteric artery, as also reported in other tissues [37]. However, although tranilast did not affect NA release, it did reduce the contractile response, as observed with other vasoconstrictor agents [18,38]. This effect can be attributed to inhibition of the Ca^2+^ influx from the extracellular environment and Ca^2+^ release from intracellular Ca^2+^ stores, as previously reported [39,40]. The fact that vasoconstriction produced by KCl remained unmodified in the presence of tranilast suggests that this drug interferes with the Ca^2+^ movement coupled to receptor activation, but not to the movement induced by depolarization. This differential influence on adrenergic innervation seems to be implicated in the opposite results obtained when incubating with one mast cell stabilizer or the other.

After preincubation with phentolamine, we observed a remnant contractile response to EFS in control and ketotifen-incubated segments, but not after tranilast treatment. This result indicates that tranilast abolished the function of another vasoconstrictor factor. The depletion of sympathetic innervation in control and ketotifen treated segments by preincubation with the neurotoxin 6-OHDA abolished the remant vasoconstriction, thereby confirming that this contractile neurotransmitter has a sympathetic origin in control and ketotifen-incubated segments. This neurotransmitter could probably be ATP, as we have previously observed in other experimental conditions [21,30].

Taken together, these results indicate that both ketotifen and tranilast alter the vasoconstrictor response to EFS in rat mesenteric artery, but in an opposite manner: ketotifen increases EFS-induced contraction, while tranilast decreases it. This fact is due to different actions by these drugs on the sympathetic and nitrergic innervations: both ketotifen and tranilast diminish nitrergic innervation function through a decrease in nNOS activation, while tranilast also decreases sympathetic innervation function, mainly through decreased NA vasoconstriction. These results indicate that the election of the mast cell stabilizer could be relevant, since it could induce important hemodynamic changes.
